# Clinical course during 40-year follow-up of Axenfeld-Rieger syndrome in a Japanese family

**DOI:** 10.4103/0974-620X.60021

**Published:** 2010

**Authors:** Shigeo Yoshida, Aki Miyazaki, Keijiro Ishikawa, Yasuhiro Ikeda, Kimihiko Fujisawa, Tatsuro Ishibashi

**Affiliations:** Department of Ophthalmology, Kyushu University Graduate School of Medical Sciences, Fukuoka, Japan

Axenfeld-Rieger Syndrome (ARS) is an autosomal dominant disorder with anterior dysgenesis of the eye; and is genetically heterogeneous. ARS patients have a high risk of blindness due to glaucoma. Three loci on chromosomes 4q25, 6p25, and 13q14 have been mapped for ARS.[1,2] Of these, mutations in the forkhead winged/helix transcription factor, FOXC1 on chromosome 6p25[3] and the homeodomain protein PITX2 on chromosome 4q25[4] have been implicated as the cause of ARS. The diagnosis of ARS is made by a constellation of ocular findings, including excessive iris tearing (polycoria), iris hypoplasia, eccentric pupils, prominent and displaced Schwalbe′s line (posterior embryotoxon), and iridocorneal tissue adhesions. Extraocular developmental abnormalities, especially of the teeth, facial bones, and periumbilical skin, have also been reported to be associated with ARS.[5] Although the ARS-associated characteristics have been well-documented, the long-term follow-up of the clinical course of ARS has been rarely reported. We report the clinical course of ARS during a 40 year follow-up in a Japanese patient.

**Figure 1:**
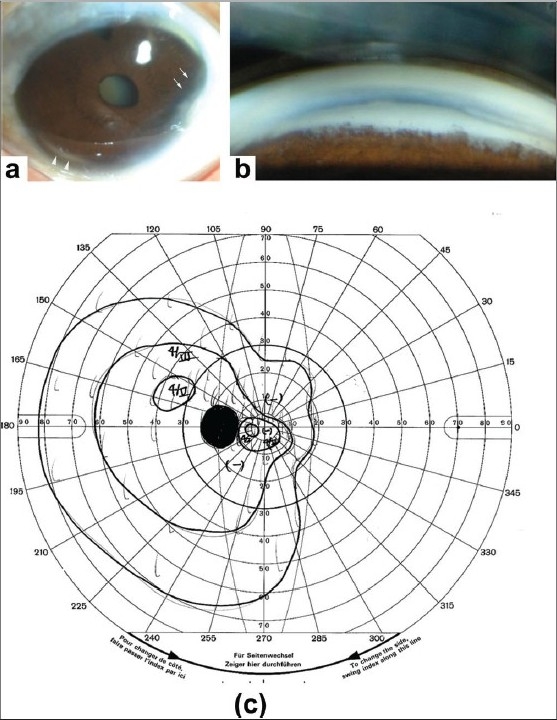
Photographs of the anterior segment of a 42-year-old woman with Axenfeld- Rieger syndrome. (a) Anterior segment photographs of the left eye of the proband at the age of 42 years showing microcornea, iris hypoplasia (arrows), and posterior embryotoxon (arrowheads). Deposits are seen at the corneal limbus which may reflect aging changes. Nuclear cataracts were also present. (b) Gonioscopic images of the same patient showing an abnormal angle and iridocorneal tissue adhesions traversing the anterior chamber. (c) Goldmann kinetic perimetric fields of the patient at the age of 42 years

The proband was a 42-year-old Japanese woman. When she first visited our clinic at two years of age, she had microcornea (corneal diameter: 9 mm OU), iris hypoplasia, and posterior embryotoxon by slit-lamp examination. The intraocular pressure (IOP) was 18 mmHg OD and 30 mmHg OS. Gonioscopy demonstrated iridocorneal tissue adhesions traversing the anterior chamber. Because of the high IOP OS, trabeculectomy was immediately performed, and the IOP decreased to 17 and 20 mmHg OU. Her visual acuity was documented as 20/20 OU at 4-years-of-age. Since then, although her IOPs were recorded at 16 mmHg with topical 0.5% betaxolol and/or 0.005% latanoprost, the IOPs occasionally increased to around 25 mmHg OU. Anterior segment photographs of the proband at the age of 42 years are shown in Figure 1a and b. At 42, her visual acuity had decreased 20/100 OD because of nuclear cataract. She also developed optic nerve damage with glaucomatous visual field loss in the left eye [Figure 1c]. She is continuing to be followed.

**Figure 2:**
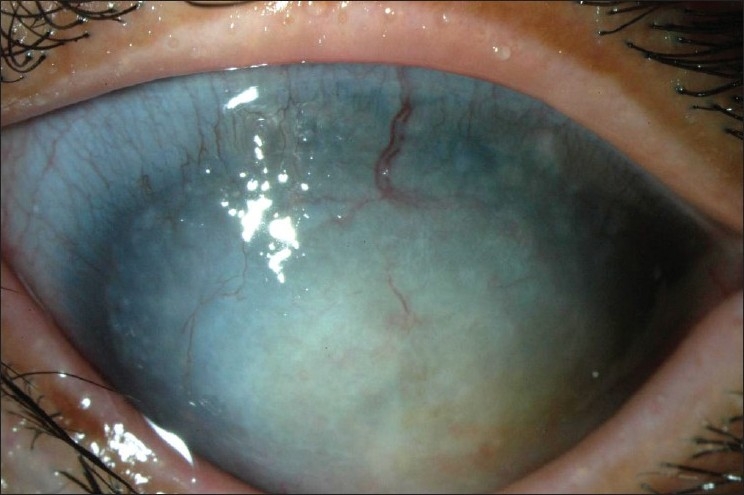
Photograph of anterior segment of the left eye of the proband's son at the age of 9 years showing a corneal opacity with a mulberry-like appearance. Corneal neovascularization can also be seen

The proband′s 12-year-old son, who was the product of a normal pregnancy and delivery, first visited our clinic when he was 1.5-years-of-age. He was not able to visit us earlier because his family had temporarily moved to another district of Japan. His parents reported that he developed corneal opacities OU soon after birth. On initial examination, in our hospital, he exhibited buphthalmus with elevated IOPs of 36 and 40 mmHg OU. Slit-lamp examination demonstrated bilateral central corneal stromal opacity, posterior embryotoxon, an eccentric pupil, and iridocorneal tissue adhesions in both eyes. Combined trabeculotomy-trabeculectomy OD and trabeculectomy OS were immediately performed, and the IOP decreased to 17 mmHg OD and 22 mmHg OS, respectively. His visual acuity was 0.02 OD and 0.06 OS at three years of age, but the IOPs progressively increased to 30 mmHg, and his visual acuity decreased to hand movements OU by the age of nine years because of a worsening of the corneal opacities [Figure 2].

No systemic developmental defects were identified in both the proband and her son. Based on the family history and characteristic anterior segment dysgenesis, a diagnosis of ARS was made. Although we performed trabeculectomy at a younger age in the son than in the mother, the surgical intervention did not preserve vision very well in the son′s eyes. The central corneal stromal opacity observed in the son but not in the mother, was perhaps due to edema associated with permeability of the endothelial barrier. This indicated that the disease process was in an advanced stage. This is consistent with the observations by Kawase *et al*. that in three of the four pedigrees, the sons of women with the same FOXC1 mutation had more severe glaucoma than their mothers.[6] This prompted us to perform molecular genetic analysis with informed consent.[7]

The direct sequencing of *FOXC1* and *PITX2*, however, did not reveal any disease-causing mutations in both genes, suggesting that either the mutation was located outside the exonic region of those genes, or there was a micro-deletion within the genes. It is also possible that the ARS in our patients was linked to13q14 locus or others as yet unknown ARS-causing genes. The different clinical courses of two cases of ARS may be due to the complex interactions of several genes involved in the formation of the anterior segment. Nevertheless, the clinical course of the mother indicates that visual function can be achieved possibly with early surgical intervention.
